# Neural crest-mediated bone resorption is a determinant of species-specific jaw length

**DOI:** 10.1016/j.ydbio.2015.10.001

**Published:** 2015-10-21

**Authors:** Erin L. Ealba, Andrew H. Jheon, Jane Hall, Camille Curantz, Kristin D. Butcher, Richard A. Schneider

**Affiliations:** a Department of Orofacial Sciences, University of California, San Francisco, USA; b Department of Orthopaedic Surgery, University of California, San Francisco, USA

**Keywords:** Avian beak development and evolution, Jaw length, Cranial neural crest, Bone resorption, Bone remodeling, Quail-duck chimeras, Quck, Osteoclasts, Osteocytes, Osteocytic osteolysis, TRAP staining, Mmp9, Mmp13, OPG, RANKL, Bisphosphonates, Bone mineral density, Evolutionary developmental biology

## Abstract

Precise control of jaw length during development is crucial for proper form and function. Previously we have shown that in birds, neural crest mesenchyme (NCM) confers species-specific size and shape to the beak by regulating molecular and histological programs for the induction and deposition of cartilage and bone. Here we reveal that a hitherto unrecognized but similarly essential mechanism for establishing jaw length is the ability of NCM to mediate bone resorption. Osteoclasts are considered the predominant cells that resorb bone, although osteocytes have also been shown to participate in this process. In adults, bone resorption is tightly coupled to bone deposition as a means to maintain skeletal homeostasis. Yet, the role and regulation of bone resorption during growth of the embryonic skeleton have remained relatively unexplored. We compare jaw development in short-beaked quail versus long-billed duck and find that quail have substantially higher levels of enzymes expressed by bone-resorbing cells including tartrate-resistant acid phosphatase (TRAP), *Matrix metalloproteinase 13* (*Mmp13*), and *Mmp9*. Then, we transplant NCM destined to form the jaw skeleton from quail to duck and generate chimeras in which osteocytes arise from quail donor NCM and osteoclasts come exclusively from the duck host. Chimeras develop quail-like jaw skeletons coincident with dramatically elevated expression of TRAP, *Mmp13*, and *Mmp9*. To test for a link between bone resorption and jaw length, we block resorption using a bisphosphonate, osteoprotegerin protein, or an MMP13 inhibitor, and this significantly lengthens the jaw. Conversely, activating resorption with RANKL protein shortens the jaw. Finally, we find that higher resorption in quail presages their relatively lower adult jaw bone mineral density (BMD) and that BMD is also NCM-mediated. Thus, our experiments suggest that NCM not only controls bone resorption by its own derivatives but also modulates the activity of mesoderm-derived osteoclasts, and in so doing enlists bone resorption as a key patterning mechanism underlying the functional morphology and evolution of the jaw.

## 1. Introduction

Bird beaks are among the most exquisitely adapted and highly diversified structures of vertebrates. Beaks seem perfectly suited for intricate species-specific behaviors related to feeding, predation, vocalization, mating, and preening. Such fundamental connections between form and function served as the foundation for early theories of evolution by natural selection best epitomized by the beaks of Darwin’s finches (Darwin, 1859). Ever since Darwin, the majority of work on beak evolution has focused on the role of the ecological niche and behavior in shaping the beak. In contrast, much less attention has been given to molecular and cellular mechanisms that generate species-specific differences in beak morphology.

**Fig. 1:**
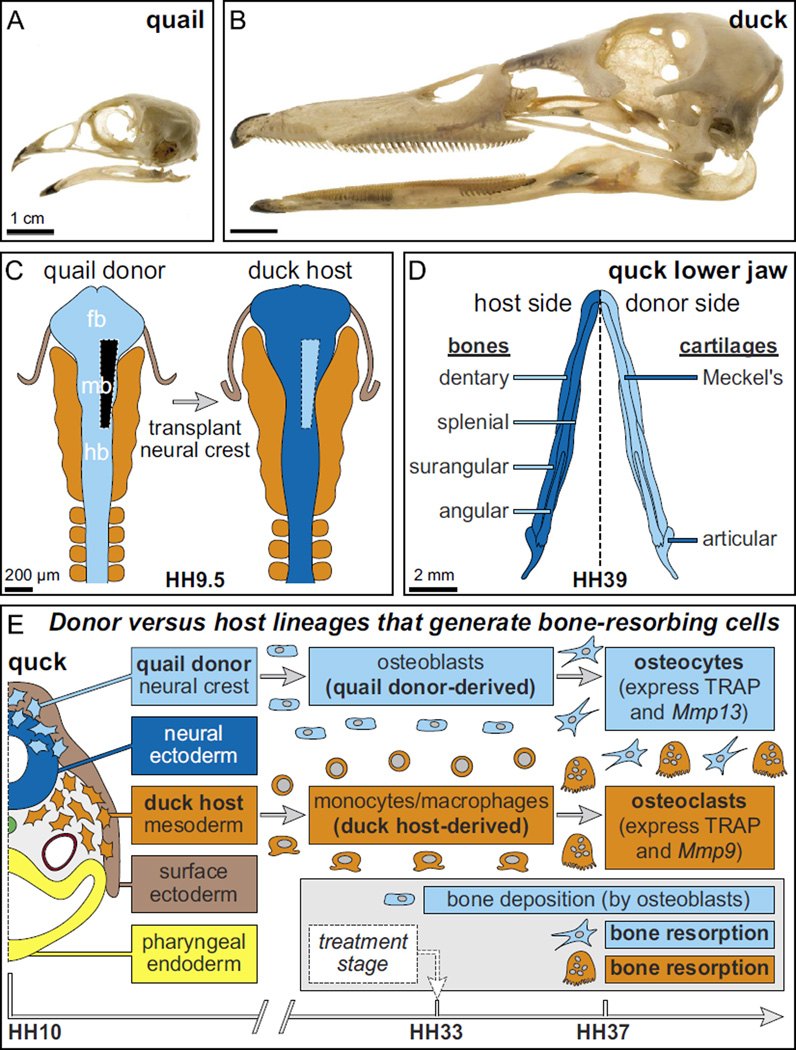
Species-specific jaw length, the quail-duck chimeric system, and lineages that resorb bone. Adult (A) quail and (B) duck skulls exhibit species-specific differences in jaw length. (C) Neural crest mesenchyme (NCM) that gives rise to the jaw skeleton is excised unilaterally from the boundary between the forebrain (fb) and midbrain (mb) to the rostral hindbrain (hb) of a quail donor (light blue) and transplanted orthotopically into a duck host (dark blue) at Hamburger and Hamilton (HH) stage 9.5 to make chimeric quck. (D) Schematic of a chimeric quck lower jaw in dorsal view at HH39 showing quail donor-derived bones and cartilages on one side (light blue) and duck host-derived bones and cartilages on the other (dark blue). Almost all of the lower jaw bones form through intramembranous ossification and these are the dentary, splenial, surangular, and angular. The articular cartilage undergoes endochondral ossification whereas Meckel’s cartilage remains unossified. (E) Schematic of an HH10 chimeric quck embryo in a transverse section plane through the caudal midbrain showing embryonic precursor populations that participate in the deposition and resorption of bone in the jaw skeleton. Specifically, osteoblasts and osteocytes are derived from quail donor NCM (light blue), while osteoclasts are derived from duck host mesoderm (orange). Osteoblasts begin depositing bone by HH33 whereas osteocytes and osteoclasts participate in bone resorption starting around HH37. In the lower jaw of quck chimeras, tartrate-resistant acid phosphatase (TRAP) is expressed by both quail donor-derived osteocytes and duck host-derived osteoclasts whereas *Mmp13* is expressed by osteocytes, *Mmp9* is expressed by osteoclasts, and neither is expressed by chondrocytes in Meckel’s cartilage since there is no endochondral ossification. Based on this timeline, treatments to inhibit or activate bone resorption were administered at HH33.

By creating chimeras of Japanese quail and white Pekin duck (Fig. 1(A and B)) we have demonstrated that ultimately, the species-specific size and shape of the beak is governed by cranial neural crest mesenchyme (NCM). NCM originates along the dorsal margins of the neural tube and generates a variety of tissues including bone and cartilage of the beak (Jheon and Schneider, 2009; Noden and Schneider, 2006). When NCM is transplanted between quail and duck, quail NCM gives rise to short, blunt quail-like beaks on duck hosts (“quck”), whereas duck NCM produces long, broad duck-like bills on quail hosts (“duail”) (Schneider, 2005; Schneider and Helms, 2003). Transplanting NCM unilaterally (Fig. 1(C)) allows donor cells to fill one side of the host jaw skeleton (Fig. 1(D)), maintains the non-surgical side as an internal control, and facilitates a direct comparison of donor- and host-derived tissue in the same chimeric skeleton (Eames and Schneider, 2005, 2008; Fish and Schneider, 2014a; Lwigale and Schneider, 2008; Solem et al., 2011; Tokita and Schneider, 2009; Tucker and Lumsden, 2004). Quail embryos develop considerably faster than do duck, and so quail NCM when transplanted into a slower-developing duck follows its intrinsic rate of maturation and executes molecular and cellular programs approximately three stages ahead of the duck host (Eames and Schneider, 2005, 2008; Hall et al., 2014; Lwigale and Schneider, 2008; Merrill et al., 2008; Schneider and Helms, 2003). This offers a way to evaluate the effects of donor cells on the host by looking for species-specific changes to the timing of gene expression and/or cell differentiation. There is also an anti-quail antibody (Q¢PN) that does not recognize duck cells and permits donor and host contributions to be distinguished from one another. Furthermore, we can assess the proportion of quail versus duck on the molecular level using a PCR-based strategy (Ealba and Schneider, 2013).

Much remains to be understood about how NCM accomplishes the complex task of establishing species-specific pattern in the facial skeleton, and what particular mechanisms function as determinants of beak length. In this regard, the quail-duck chimeric system has been useful for illuminating critical events and signaling interactions involving donor NCM and host tissues in a relatively normal developmental context, and has led us to conclude that NCM employs a series of precise mechanisms to control species-specific size and shape. First, during the migration and allocation of NCM, duck have more progenitors destined to form the jaw skeleton than do quail; second, when these progenitor populations expand, there is species-specific regulation of, and response to, multiple signaling pathways including Sonic Hedgehog (SHH), Fibroblast Growth Factor (FGF), and Bone Morphogenetic Protein (BMP); and third, as these progenitors differentiate into the jaw skeleton, they execute autonomous molecular and cellular programs that control the development of cartilage and bone in a manner that is intrinsic to each species (Eames and Schneider, 2008; Fish et al., 2014; Hall et al., 2014; Merrill et al., 2008; Mitgutsch et al., 2011; Schneider, 2015; Schneider and Helms, 2003).

In the current study, we have discovered that NCM also mediates a previously unrecognized but correspondingly fundamental mechanism that regulates jaw length, which is the process of bone resorption. Two cell types actively resorb bone. Mesoderm-derived osteoclasts have been regarded as the predominant cells that resorb bone during remodeling (Boyle et al., 2003; Filvaroff and Derynck, 1998; Hancox, 1949; Martin and Ng, 1994; Teitelbaum, 2000; Teitelbaum et al., 1997). In our chimeric system, osteoclasts come exclusively from the host (Fig. 1(E)) (Jotereau and Le Douarin, 1978; Kahn et al., 2009). However osteocytes, which in the beak skeleton arise solely from NCM (Helms and Schneider, 2003; Le Lièvre, 1978; Noden, 1978), also resorb bone (Belanger, 1969; O’Brien et al., 2008; Qing et al., 2012; Tang et al., 2012; Xiong and O’Brien, 2012; Xiong et al., 2014). Here we discover that osteocytes and osteoclasts express species-specific molecular programs associated with the resorption of bone, and we establish that these programs are controlled by NCM. Then, using a gain- and loss-of-function strategy, we target these programs and find that bone resorption is mechanistically linked to beak length, especially in the lower jaw. Such results build upon other studies that have implicated calmodulin signaling, which is known to regulate osteocyte and osteoclast activity (Choi et al., 2013a; Choi et al., 2013b; Seales et al., 2006; Zayzafoon, 2006), in establishing species-specific beak length (Abzhanov et al., 2006; Gunter et al., 2014; Schneider, 2007). Finally, we show that NCM also directs a parallel role for bone resorption, which is the establishment of species-specific bone mineral density (BMD). Taken together, our study suggests that bone resorption is an additional mechanism that operates at later stages in development through which NCM controls the morphological evolution of the avian beak.

## 2. Materials and methods

### 2.1. The use of avian embryos

Fertilized eggs of Japanese quail (*Coturnix coturnix japonica*) and white Pekin duck (*Anas platyrhynchos*) were purchased from AA Lab Eggs (Westminster, CA) and incubated at 37 °C in a humidified chamber until they reached embryonic stages appropriate for manipulations, treatments, and analyses. For all procedures, we adhered to accepted practices for the humane treatment of avian embryos as described in S3.4.4 of the *AVMA Guidelines for the Euthanasia of Animals: 2013 Edition* (Leary et al., 2013).

Embryos were matched at equivalent stages using an approach that is based on external morphological characters and that is independent of body size and incubation time (Hamilton, 1965; Ricklefs and Starck, 1998; Starck and Ricklefs, 1998). The Hamburger and Hamilton (HH) staging system, originally devised for chick, is a well-established standard (Hamburger and Hamilton, 1951). Separate staging systems do exist for duck (Koecke, 1958) and quail (Ainsworth et al., 2010; Nakane and Tsudzuki, 1999; Padgett and Ivey, 1960; Zacchei, 1961) but these embryos can also be staged via the HH scheme used for chicken (Ainsworth et al., 2010; Le Douarin et al., 1996; Lwigale and Schneider, 2008; Mitgutsch et al., 2011; Schneider and Helms, 2003; Smith et al., 2015; Starck, 1989; Yamashita and Sohal, 1987; Young et al., 2014). Criteria utilized to align quail and duck at a particular HH stage change over time depending on which structures become prominent. For early embryonic stages, we used the extent of neurulation, neural crest migration, and somitogenesis as markers (Fish et al., 2014; Lwigale and Schneider, 2008; Schneider and Helms, 2003); whereas later, we relied on growth of the limbs, facial primordia, feather buds, and eyes since these become more diagnostic (Eames and Schneider, 2005; Merrill et al., 2008).

### 2.2. Generation of chimeras

Quail and duck eggs were windowed at HH9.5 using surgical scissors and transparent tape. Embryos were visualized by application of Neutral Red (Sigma) with a blunt glass rod. At HH9.5, NCM is most abundant along the dorsal midline of the anterior neural tube (Tosney, 1982). Hand-made, flame-sharpened tungsten needles and Spemann pipettes were used for surgical operations (Fish and Schneider, 2014a; Lwigale and Schneider, 2008; Schneider, 1999; Schneider and Helms, 2003). Bilateral and unilateral grafts of rostral hindbrain and midbrain NCM were made from quail to duck (“quck”) or duck to quail (“duail”) (Lwigale and Schneider, 2008; Schneider and Helms, 2003). Donor tissue was inserted into a host that had comparable regions of tissue removed. For controls, orthotopic grafts or sham operations were made within each species and were equivalent to those performed in previous studies (Noden, 1983; Schneider, 1999; Schneider and Helms, 2003; Schneider et al., 2001). Controls were incubated alongside chimeras in order to ensure that the stages of grafted cells in the donor, host, and chimeras were accurately assessed. After surgery, eggs were closed with tape and incubated until reaching stages appropriate for additional experimental manipulation or analysis.

### 2.3. Histology and immunohistochemistry

Quail, duck, quck, and duail embryos were collected at HH35–HH40 in either 4% paraformaldehyde (PFA) or Serra’s fixative overnight at 4 °C (Schneider, 1999). To detect tartrate-resistant acid phosphatase (TRAP), quail, duck, and quck embryos were stained in whole-mount using the Acid Phosphatase Leukocyte kit (Sigma) following the manufacturer’s protocol, except 7 mg/mL Fast Red Violet were used in place of the Fast Garnet GBC Base Solution. To detect TRAP in sections, embryos were dehydrated, embedded in paraffin, and cut into 7 µm sagittal sections. Slides were deparaffinized and rehydrated, and TRAP staining was performed (as described above) in a humidified chamber at 37 °C. To detect quail donor cells, sections from chimeric quck embryos were immunostained with the quail nuclei-specific Q¢PN antibody (Developmental Studies Hybridoma Bank, Iowa City, IA, USA) (Schneider, 1999). To detect bone, cartilage, and surrounding tissue, sections were differentially stained with Milligan’s trichrome (Eames and Schneider, 2005; Hall et al., 2014; Presnell and Schreibman, 1997; Solem et al., 2011; Tokita and Schneider, 2009). For whole-mount skeletal preparations, embryos were stained with Alizarin red, and cleared in glycerol (Hall et al., 2014; Mitgutsch et al., 2011; Wassersug, 1976).

### 2.4. Three-dimensional reconstructions and volumetric data

Quail, duck, and quck mandibles were collected at HH37 and cut into 10 µm coronal sections (i.e., in the horizontal plane of the mandible). Adjacent sections stained for either osteoid or TRAP at 80 µm intervals were imaged and merged using Adobe Photoshop. Images were imported into the Amira 6 3D Software package (FEI, Hillsboro, OR) and aligned manually using anatomical landmarks including Meckel’s cartilage and the epithelium along the oral cavity. After image slices were aligned for each specimen, then the areas of interest (osteoid, TRAP, and Meckel’s cartilage) were manually segmented into data objects (image voxels) using a range of segmentation tools. Once all areas of interest were labeled for each specimen, 3D models were generated and volumetric data for TRAP and osteoid were extracted. For each quail and duck specimen, the data were represented as the average volume of the left and right sides of the mandible. For quck specimens, data from the donor and host sides were extracted separately.

### 2.5. Gene expression analyses

To analyze stage- and species-specific levels of gene expression, reverse transcription quantitative PCR (RT-qPCR) was performed (Bustin et al., 2009). Total RNA was isolated from quail, duck, and chimeric quck mandibles (with the tongue removed) at HH34 and HH37 using an RNeasy column purification kit (Qiagen, Valencia, CA) as described previously (Ealba and Schneider, 2013; Fish et al., 2014; Hall et al., 2014). Concentration and purity of RNA were assessed using a Nanodrop ND-1000 (Thermo Scientific, Wilmington, DE). Approximately 250 ng of total RNA was converted to cDNA in a 20 µl reverse transcription reaction using 1 µl of iScript reverse transcriptase (Bio-Rad, Hercules, CA). The reaction involved: step 1, 25 °C for 5 min; step 2, 42 °C for 30 min; step 3, 85 °C for 5 min; step 4, 4 °C hold in a 2720 Thermal Cycler (Applied Biosystems, Carlsbad, CA).

RT-qPCR was performed in a C1000 Thermal Cycler with a CFX96 Real–Time System (Bio-Rad). Forward and reverse primers, 2 µl of cDNA, RNase-free dH20, and iQ SYBR-Green Supermix (Bio-Rad), containing dNTPs, iTaq DNA polymerase, MgCl2, SYBR Green I, enhancers, stabilizers, and fluorescein, were manually mixed in a 25 µl reaction to amplify each cDNA of interest. Samples were run in triplicate on white hard-shell 96-well PCR plates (Bio-Rad). The protocol was: step 1, 95 °C for 3 min; step 2, 95 °C for 10 s; step 3, 60 °C for 30 s and a plate read; steps 2 and 3 were repeated 39 times; step 4, 95 °C for 10 s; step 5, melt curve of 60–90 °C for 5 s at each 0.5 °C with a plate read. Melt curves were checked for specificity. PCR products amplified after 35 cycles were considered to be false positives. Primers were designed using NCBI Primer-BLAST software for the following chicken genes: *Collagen type 1 alpha 1* (*Col1α1*) (Forward 5′-CCCGACCCTAAGACAAAGAG-3′ and Reverse 5′-GCTACTTACTGTCCTCTTCTCC-3′) with an amplicon length of 143bp; *Matrix Metalloproteinase 13 (Mmp13)* (Forward 5′-GCTGGAGACAGAGATCCCAACCCA-3′ and Reverse 5′-GCGGGTGCAGTCGCCAGAAA-3′) with an amplicon length of 139 bp; and *Matrix Metalloproteinase 9 (Mmp9)* (Forward 5′-CGGCAGCCAAGAGCATGGTGA-3′ and Reverse 5′-AGCTGGCCCCGTTGGCATTC-3′) with an amplicon length of 179bp). Expression levels for *Col1α1, Mmp13*, and *Mmp9* were normalized to expression of the reference gene *Ribosomal protein L19* (*RPL19*), (Forward 5′-ACGCCAACTCGCGTCAGCAG-3′ and Reverse 5′-ATATGCCTGCCCTTCCGGC-3′, with an amplicon length of 127 bp) (Ealba and Schneider, 2013). Expression was checked to make sure amplification efficiencies were equal among samples. Fold changes were calculated using the delta-delta C(t) method (Livak and Schmittgen, 2001). To assess relative fold changes between stages, duck at HH37 was compared to duck at HH34, which was set to one; quail at HH37 to quail at HH34, which was set to one; and quck at HH34 to quail at HH34, which was set to one.

Prior to analyzing genes of interest in chimeric quck, cases were pre-screened by applying a previously published strategy for estimating the percentages of donor versus host cells using species-specific primers to RPL19 (Ealba and Schneider, 2013). Chimeric samples were included in the analysis only if they contained greater than 60% of quail donor-derived cells in the mandible.

To assay for spatial and temporal patterns of gene expression, *in situ* hybridization analyses were performed on paraffin sections as described (Albrecht et al., 1997; Schneider, and Helms, 2003). Quail, duck, and quck sections were hybridized with ^35^S-labeled antisense riboprobes to chicken *Mmp13* and *Mmp9*. *S*ections were counterstained with a blue nuclear stain (Hoechst Dye; Sigma). Hybridization signals were detected using illuminated darkfield and the nuclear stain was visualized using epifluorescence.

### 2.6. Inhibiting and activating resorption

Ten microliters of alendronate sodium trihydrate (0.9 µg/µl) (Sigma, A4978), recombinant mouse osteoprotegerin (rOPG) protein (200 ng/µl) (R&D Systems, Inc., 459-MO-100), or recombinant mouse RANK Ligand (rRANKL) protein (40 ng/µl) (Sigma, R0525), were injected into the vitelline vein of quail and duck at HH33 using glass needles (diameter 0.5 mm, Sutter Instruments Co.), and a PV830 Pneumatic Picopump (World Precision Instruments, Sarasota, FL). Concentrations were determined following dose-response studies and published literature. Five microliters of MMP13 inhibitor (2 µg/µl) (EMD Millipore, Inc., 444283) were administered onto the chorioallantoic membrane of HH33 embryos directly above the head (this approach was used instead of vitelline vein injections, which caused embryonic lethality). Control embryos were treated with 0.1% bovine serum albumin (BSA) or dimethylsulfoxide (DMSO). Embryos were collected at HH38 in PFA.

### 2.7. Measurement of jaw length

Using *ImageJ* (NIH) on specimens imaged in lateral view, lower jaw measurements were made from the proximal tip of the angular bone to the distal tip of the dentary bone. Upper jaw measurements were taken from the tip of the nasal bone in the center of the maxilla to the distal tip of the premaxilla. Lower jaw measurements were divided by upper jaw measurements to normalize for size and the values were presented as ratios.

### 2.8. Analysis of bone mineral density

Bone mineral density (BMD) of adult quadratojugal/jugal and mandible bones were measured by Dual-energy X-ray Absorptiometry (DXA) using a PIXImus instrument in ultrahigh resolution mode (Lunar, France; Software version 1.44). BMD of the lower jaw in embryonic quail, duck, and quck was measured using a SCANCO µCT scanner (UCSF Radiology µCT core).

### 2.9. Statistics

Numerical data are represented as the mean ± either the standard deviation or standard error of the mean (as indicated) and compared by unpaired Student’s *t* test.

## 3. Results and discussion

### 3.1. NCM mediates stage- and species-specific levels of bone resorption

**Fig. 2:**
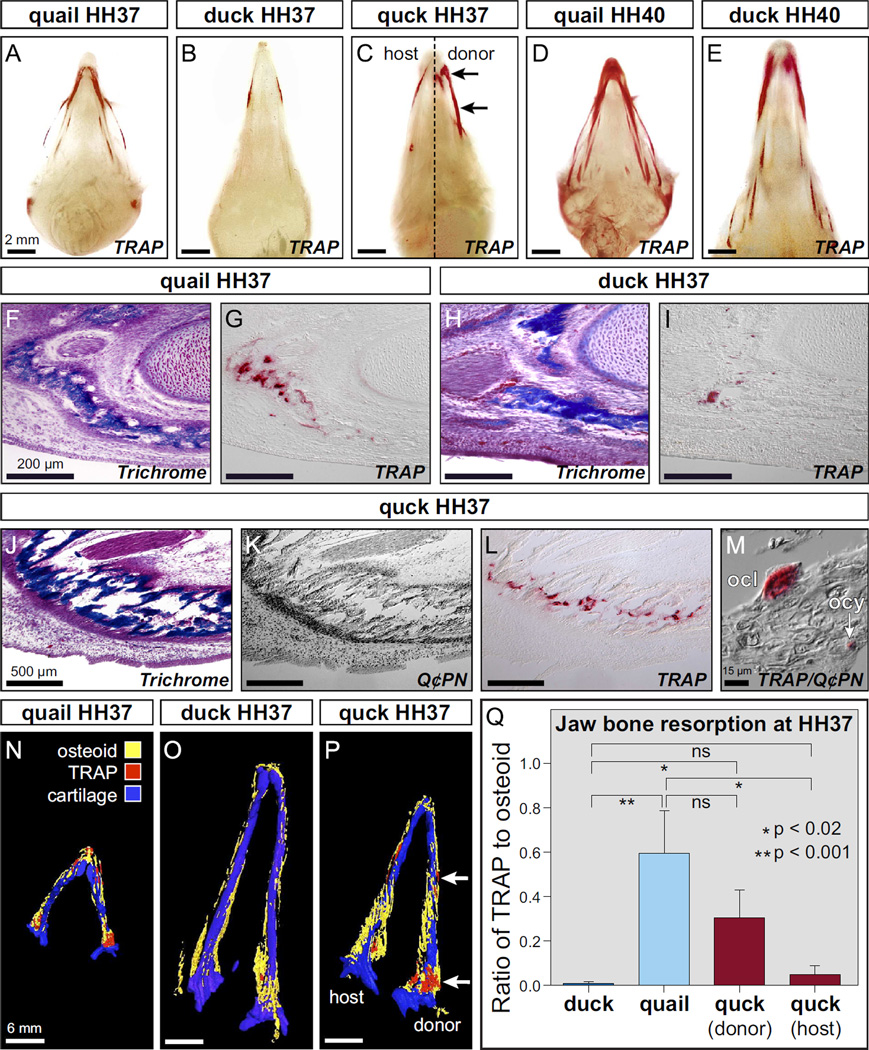
Species-specific differences in TRAP activity are mediated by NCM. (A) Whole mount TRAP staining in the head skeleton of quail (*n*=4) versus, (B) duck (*n*=4) reveals species-specific differences in levels and spatial domains of bone resorption at HH37, especially at the distal and proximal regions of the jaw. (C) Quck demonstrate that NCM controls bone resorption as indicated by higher quail-like levels and spatial patterns of TRAP activity on the donor side (arrows) of duck hosts (*n*=8). (D) By HH40, TRAP staining is more robust and widespread in quail (*n*=4) versus (E) duck (*n*=6) demonstrating both species- and stage-specific regulation of bone resorption. (F) Sections through the distal lower jaw skeleton at HH37 demonstrate that in the osteoid matrix of developing bone (stained blue), quail (*n*=6) have (G) high levels of TRAP-positive cells (stained red). (H) In equivalent bony regions of the duck jaw (I) less TRAP-positive staining can be observed (*n*=6). **(J)** Sections through the lower jaw of chimeric quck reveal that (K) coincident with the presence of quail-derived bones (Q¢PN-positive donor cells stained black) (L) there are quail-like levels of TRAP-positive staining (*n*=6). (M) Based on their small size and location within the bone matrix, osteocytes (ocy) can be recognized as being both TRAP-positive and Q¢PN-positive (arrow). Based on their large and irregular morphology, osteoclasts (ocl) can be identified as TRAP-positive but Q¢PN-negative. (N) 3D reconstruction of a quail mandible at HH37 showing the distribution of osteoid matrix (yellow) and TRAP staining (red) down the length of Meckel’s cartilage (blue). (O) Substantially lower levels of TRAP staining can be seen in the duck mandible. (P) The donor side of the chimeric quck mandible has higher levels of TRAP (arrows) like that observed in quail whereas the host side has lower duck-like levels. (Q) Quantification of the ratio of TRAP to osteoid volume in duck (*n*=4), quail (*n*=4), and quck (*n*=2) demonstrates that there are statistically significant differences between duck and quail in the amount of bone resorption, and that the donor side of quck is more quail-like whereas the host side is more duck-like.

To evaluate the extent to which species-specific differences in beak length might be related to the process of bone resorption, we stained whole quail and duck heads between HH35 and HH40 for TRAP, which is secreted by both osteoclasts and osteocytes (Minkin, 1982; Qing et al., 2012; Tang et al., 2012). TRAP and especially its differential expression along the surface of bone, has long been considered a marker of sites where bone is being actively resorbed (Ballanti et al., 1997; Miller, 1985; Minkin, 1982; Price et al., 1995). TRAP activity was not seen in either quail or duck at stage HH35 (data not shown) but became apparent by HH37 (Fig. 2(A, B)). TRAP levels increased by HH40 as the beak skeleton matured (Fig. 2(D, E)). At both time-points, we observed higher levels and different spatial domains of TRAP activity in quail relative to that found in stage-matched duck, signifying that quail undergo more bone resorption than duck, and suggesting that elevated resorption may relate to their shorter beaks. To test if the higher levels of bone resorption observed in quail are mediated by NCM, we analyzed TRAP activity in whole chimeric quck heads at HH37. We observed quail-like levels and spatial patterns of TRAP activity in duck hosts, indicating that NCM mediates resorption in a species-specific manner (Fig. 2(C)).

Sections through the jaw skeleton at HH37 demonstrated that in the osteoid matrix of developing bone (Fig. 2(F)), quail have higher levels of TRAP-positive cells (Fig. 2(G)) than do duck in equivalent regions of the jaw (Fig. 2(H, I)). Sections from chimeric quck revealed that quail-like levels of TRAP-positive staining were found throughout quail-derived bones of the beak skeleton (Fig. 2 (J, K and L)). Within these bones we observed TRAP-positive cells that presumably were osteocytes based on the fact that they originated from quail donor NCM (*i.e*., Q¢PN-positive), were small in size, and were located within the bone matrix. Other TRAP-positive cells presumably were osteoclasts based on the fact that they arose from the duck host (*i.e*., Q¢PN-negative), had large and irregular morphology, showed ruffled borders, and were distributed along the margins of bone matrix (Fig. 2(M)).

To visualize and quantify the amounts of osteoid staining and TRAP activity at HH37, the lower jaws of duck, quail, and quck chimeras were sectioned, stained, imaged, and reconstructed as three-dimensional (3D) projections. These analyses reveal that quail and duck exhibit species-specific differences in the levels and spatial distribution of TRAP activity, and that these differences are mediated by NCM in chimeras (Fig. 2(N–P)). By comparing volumetric data from these 3D reconstructions, we find that quail have significantly higher levels of TRAP activity than do duck (Table 1 and Fig. 2(Q)). The ratio of TRAP volume to osteoid volume is almost 75 times higher in quail relative to duck. Moreover, in chimeras the ratio of TRAP volume to osteoid volume on the donor side is much more like that observed in quail and almost 40 times higher than that observed in duck. Thus, species-specific differences in bone resorption, as represented by levels of TRAP activity, correlate with beak length and are regulated by NCM.

### 3.2. NCM regulates stage- and species-specific expression of bone resorption markers

We analyzed expression of genes known to mediate bone resorption by osteocytes and osteoclasts. *Mmp13* is expressed by osteocytes (Behonick et al., 2007; Johansson et al., 1997; Sasano et al., 2002), which in the jaw skeleton are derived entirely from NCM (Helms and Schneider, 2003; Le Lièvre, 1978; Noden, 1978), whereas *Mmp9* is expressed by osteoclasts (Engsig et al., 2000; Reponen et al., 1994), which come exclusively from mesoderm (Jotereau and Le Douarin, 1978). *Mmp13* and *Mmp9* are also expressed by hypertrophic chondrocytes when cartilage is replaced by bone during endochondral ossification (Colnot and Helms, 2001). However, during development of the lower jaw in birds, Meckel’s cartilage persists and there is no endochondral ossification except for that limited entirely to the most proximal region within the articular cartilage beginning at HH39 (Eames et al., 2004; Mitgutsch et al., 2011; Starck, 1989). All other bone in the lower jaw forms through intramembranous ossification (Helms and Schneider, 2003). Therefore, in the lower jaw of quck chimeras prior to HH39, we would expect *Mmp13* to be expressed by quail donor-derived cells (e.g., osteocytes), *Mmp9* to be expressed by duck host-derived osteoclasts, and neither to be conspicuously expressed by chondrocytes.

**Fig. 3:**
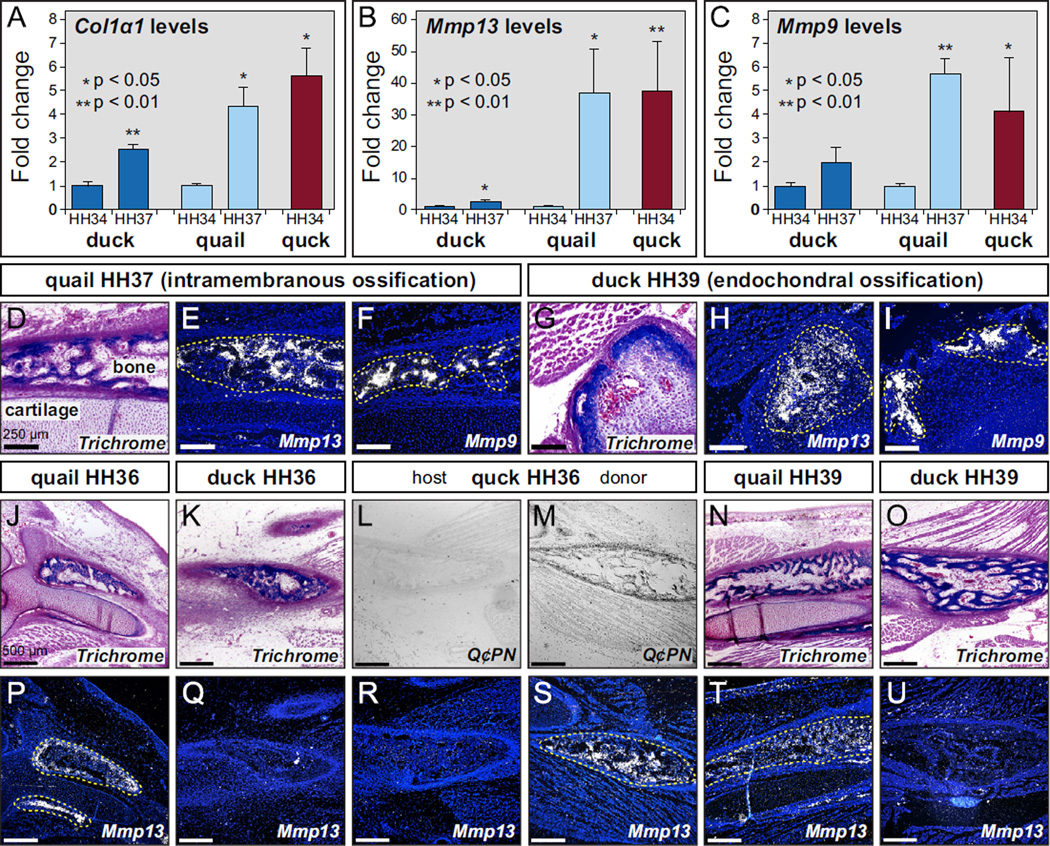
NCM controls expression of genes involved in bone resorption. (A) Using RT-qPCR to assay for *Col1*α*1* mRNA as a marker for bone deposition shows a 2.5-fold increase in duck (dark blue) from HH34 (*n*=5) to HH37 (*n*=5) and 4-fold increase in quail (light blue) from HH34 (*n*=5) to HH37 (*n*=5). Levels of *Col1*α*1* expression in chimeric quck (red) at HH34 (*n*=2) are like that observed in quail controls at HH37. (B) Levels of *Mmp13* in duck show a 3-fold increase from HH34 to HH37, whereas quail have an approximately 35-fold increase in expression from HH34 to HH37. In chimeric quck at HH34, quail donor cells maintain their higher stage-specific and species-specific levels of expression with an approximately 37-fold increase in *Mmp13*. (C) There is no significant increase in *Mmp9* expression from HH34 to HH37 in duck whereas quail have a 6-fold increase. In quck, there is a 4-fold increase in *Mmp9* at HH34 like that observed in quail at HH37. *P*-values: quail at HH37 compared to HH34; duck at HH37 to HH34; and quck at HH34 to HH34. (D) Sagittal sections showing the dentary bone and Meckel’s cartilage in the lower jaw of quail at HH37. (E) Through *in situ* hybridization on adjacent sections and coincident with the intramembranous ossification of bone, *Mmp13* transcripts can be observed in the osteoid matrix (yellow dashed line), but not in Meckel’s cartilage. (F) *Mmp9* expression is localized to discrete domains in bone presumably coincident with the distribution of osteoclasts. *Mmp9* is not detected in Meckel’s cartilage. (G) Endochondral ossification of cartilage is restricted to the proximal-most region of the duck lower jaw at HH39. (H, I) *Mmp13* and *Mmp9* are expressed in cartilage undergoing endochondral ossification. (J, K, N and O) Osteoid in quail and duck at HH36 and HH39. (L, M) In quck, duck host bone is Q¢PN-negative while quail donor bone is Q¢PN-positive (black cells). (P, Q, T and U) *Mmp13* is expressed at higher levels in quail versus duck at HH36 and HH39. (R) *Mmp13* is not detected on the duck host side of quck (S) but is highly expressed on the quail donor side coincident with Q¢PN-positive cells.

Reverse transcription quantitative PCR (RT-qPCR) revealed that there are significant stage- and species-specific differences in the expression of *Mmp13* and *Mmp9* in the lower jaw of quail relative to duck. First, we examined expression of *Col1*α*1* as a marker for bone deposition and observed a 2.5-fold increase in duck and 4-fold increase in quail from HH34 to HH37 (Fig. 3(A)). This stage-specific increase in expression is reflective of the timing and progression of bone deposition in quail and duck (Hall et al., 2014). Moreover, levels of *Col1*α*1* expression in chimeric quck at HH34 were like that observed in quail controls at HH37, which is consistent with the observation that quail donor NCM maintains its species-specific programs for bone formation in duck hosts (Hall et al., 2014; Merrill et al., 2008). Levels of *Mmp13* in duck showed a 3-fold increase from HH34 to HH37, but quite strikingly, quail had an approximately 35-fold increase in expression over the same stages (Fig. 3(B)). Thus, NCM-derived osteocytes significantly up-regulate *Mmp13* in a stage-specific manner, but much more so in quail. In chimeric quck at HH34, quail donor cells maintained their markedly higher stage-specific and species-specific levels of expression with an approximately 37-fold increase in *Mmp13* (Fig. 3 (B)).

For *Mmp9*, we observed no significant increase from HH34 to HH37 in duck whereas quail showed a 6-fold increase (Fig. 3(C)). Thus, mesoderm-derived osteoclasts up-regulate *Mmp9* in a stage-specific manner, but only in quail. However, in chimeric quck, even though *Mmp9*-expressing osteoclasts were derived from the duck host, we observed a 4-fold increase in *Mmp9* at HH34 like that observed in quail at HH37, indicating that donor NCM regulates the activity of osteoclasts. The finding that quail donor NCM not only maintains its intrinsic molecular program for higher *Mmp13* expression, but also up-regulates *Mmp9* in duck host-derived osteoclasts during bone resorption, points to another potential NCM-mediated mechanism through which chimeric quck acquire their shorter beaks.

Results of *in situ* hybridization on sections through the developing jaw skeleton support the RT-qPCR findings that expression of *Mmp13* and *Mmp9* become elevated at HH37, especially in quail. Coincident with the intramembranous ossification of bone (Fig. 3 (D)) we observed *Mmp13* transcripts distributed throughout the osteoid matrix (Fig. 3(E)). *Mmp9* expression was localized to discrete domains within the bone presumably coincident with the distribution of osteoclasts (Fig. 3(F)). We did not detect expression of either *Mmp13* or *Mmp9* in Meckel’s cartilage of the lower jaw at HH37 (Fig. 3(D–F)). Also, *Mmp13* and *Mmp9* were expressed at much higher levels in quail when compared to stage-matched duck (data not shown). Consistent with our RT-qPCR data showing a slight increase at HH37, we did detect expression of *Mmp13* and *Mmp9* using *in situ* hybridization in duck, but only at HH39 in the proximal-most region of the lower jaw wherever cartilage was undergoing endochondral ossification (Fig. 3(G–I)).

To identify the spatial domains underlying the stage- and species-specific increase in *Mmp13* expression observed with RT-qPCR, we stained the osteoid of jaw bone in quail, duck, and quck at HH36 and HH39 (Fig. 3(J–O)), and analyzed *Mmp13* expression in adjacent sections. At HH36, we observed *Mmp13* expression in the osteoid of quail (Fig. 3(P)) but almost none in duck (Fig. 3(Q)). Similarly, on the host-derived side of quck at HH36 (Fig. 3(L)), we also only observed very low levels of *Mmp13* expression (Fig. 3(R)). However, on the donor-derived side, coincident with the distribution of Q¢PN-positive cells (Fig. 3(M)), we observed substantially higher levels of *Mmp13* (Fig. 3(S)), like that found in quail at HH39 (Fig. 3(T)) rather than the lower levels observed in duck at HH39 (Fig. 3(U)).

### 3.3. Inhibiting and activating bone resorption affects beak length

**Fig. 4:**
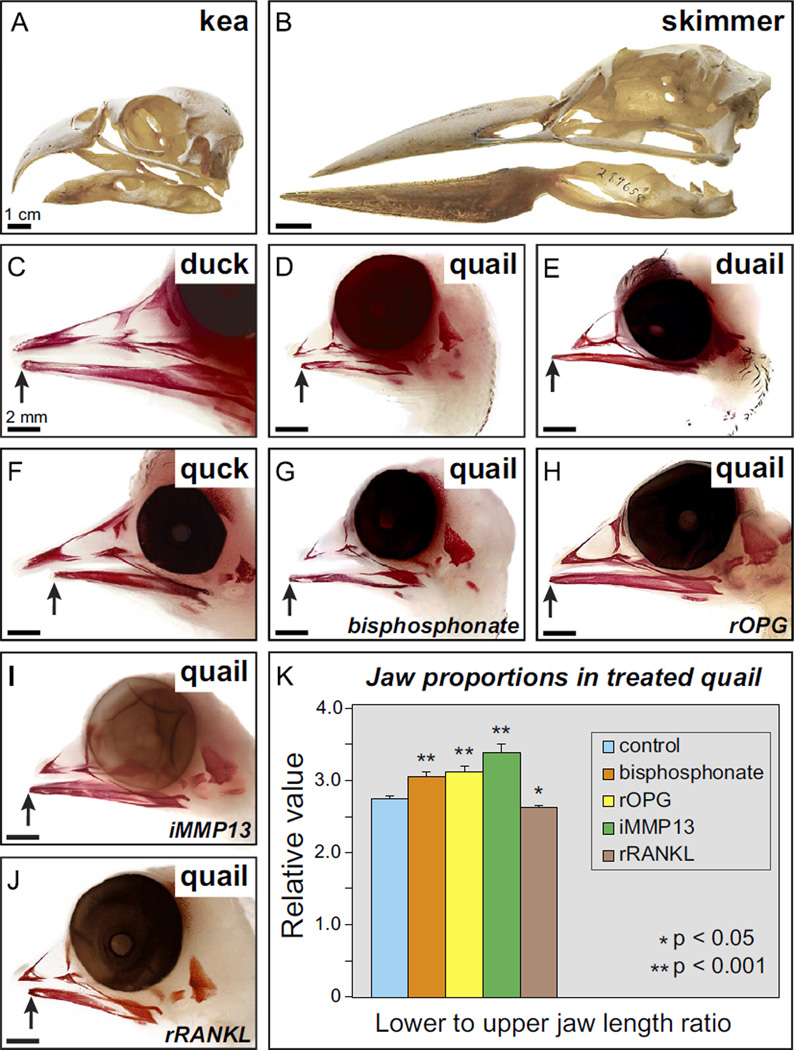
Bone resorption regulates jaw length. Differences in upper versus lower beak length occur naturally in some adult birds such as the (A) kea (*Nestor notabilis*), which has a shorter lower jaw, and (B) black skimmer (*Rynchops niger*) which has a longer lower jaw. (C and D) Quail and duck beak skeletons at HH39 stained with alizarin red showing differences in jaw length. Note the normal relations of the upper and lower portions that approximate one another at the distal tip, with the lower jaw being slightly shorter than the upper (arrows). (E) The beak is lengthened in duail when lower jaw NCM comes from a duck (*n*=4), and (F) shortened in quck when lower jaw NCM comes from a quail (*n*=7), demonstrating that NCM regulates size. (G) Quail treated at HH33 with a bisphosphonate (*n*=14), (H) recombinant OPG (rOPG) protein (*n*=8), and (I) an MMP13 inhibitor (iMMP13) have longer beaks, especially the lower jaw (*n*=6). (J) rRANKL protein treatments at HH33 decreases jaw length (*n*=8). (K) Quantifying jaw size reveals significant treatment-dependent increases and decreases in length relative to control embryos (*n*=12).

Given that quail and duck show species-specific amounts of resorption during the development of the beak, as represented by their distinct levels of TRAP staining, as well as their differential expression of *Mmp13* and *Mmp9*, we tested if changes in bone resorption can affect beak length. Typically, beak length is carefully coordinated (as in quail and duck) so that the distal tips of the upper and lower portions become aligned closely (Fig. 1(A, B)). Yet differences in upper versus lower beak length occur naturally in some adult birds such as the kea (*Nestor notabilis*), which has a shorter lower jaw (Fig. 4(A)), and black skimmer (*Rynchops niger*) which has a longer lower jaw (Fig. 4(B)). Remarkably at hatching, the upper and lower jaws of the black skimmer are equal in length, but by fledging at 4 weeks, the lower jaw is approximately 1 cm longer than the upper (Gochfeld and Burger, 1994; Hoyo et al., 1992; Zusi, 1962). Such ontogenetic and phylogenetic variation suggests that upper and lower beak lengths are regulated independently during development and point to a mechanism for fine-tuning at later stages of development and growth. Normally in the embryonic jaw skeleton of both quail and duck, the lower portion aligns with the upper aspect at the distal tip (Fig. 4(C and D)). By exchanging NCM destined to form the lower jaw skeleton between quail and duck we either lengthened the lower aspect in chimeric duail (i.e., duck donor and quail host) (Fig. 4(E)), or shortened the lower aspect in chimeric quck (i.e., quail donor and duck host) (Fig. 4(F)), indicating that upper and lower beak lengths are established separately and controlled by NCM. This is consistent with observations that fundamentally distinct molecular and cellular mechanisms operate during the patterned outgrowth of the facial primordia from which the upper and lower portions of the jaw arise (Young et al., 2000). These include regionally restricted patterns of gene expression in the populations of NCM that migrate into the upper versus lower jaw primordia, and within signaling centers in the overlying ectoderm (Abzhanov and Tabin, 2004; Ashique et al., 2002; Bhullar et al., 2015; Brugmann et al., 2007, 2010; Brunskill et al., 2014; Depew and Compagnucci, 2008; Depew et al., 1999, 2002; Doufexi and Mina, 2008; Feng et al., 2009; Fish et al., 2011; Foppiano et al., 2007; Francis-West et al., 2003; Grant et al., 2006; Havens et al., 2008; Hu and Marcucio, 2009, 2012; Hu et al., 2003, 2015a, 2015b; Jeong et al., 2004; MacDonald et al., 2004; Mina et al., 1995, 2002; Reid et al., 2011; Richman et al., 1997; Rowe et al., 1992; Schneider et al., 1999, 2001; Szabo-Rogers et al., 2008; Tavares et al., 2012; Trumpp et al., 1999; Wu et al., 2006; Young et al., 2010, 2014). Moreover, the upper and lower portions of the jaw are not equally susceptible to mutations in numerous genes or various teratogenic agents (Brown et al., 1997; Grant et al., 1997; McGonnell et al., 1998; Schilling 1997, Schneider et al., 2001; Tamarin et al., 1984), again reinforcing the notion that their outgrowth is mediated through distinct mechanisms.

To test the extent to which bone resorption is a determinant of jaw length, we used a biochemical strategy to either activate or inhibit resorption by osteocytes and/or osteoclasts, and then we assayed for changes in beak length. Treatments were administered systemically at HH33 when bone deposition is just starting and resorption has not yet begun (Fig. 1(E)). Inhibiting resorption with a bisphosphonate that is a potent inhibitor of bone resorption (Fig. 4(G)), with recombinant OPG protein (Fig. 4(H)), or with an MMP13 inhibitor (Fig. 4(I)), results in elongation of the quail beak skeleton, especially the lower jaw. In contrast, activating resorption using recombinant RANKL protein significantly shortens the beak (Fig. 4(J)). These changes in beak proportion are statistically significant in treated quail (Fig. 4(K)) and are observed despite the fact that we simply generated a ratio between lower and upper jaw length. Thus, we may be underestimating the actual amount of change in beak length if the upper jaw was also similarly affected by our treatments. Furthermore, we did not observe significant changes in jaw length when we treated duck embryos with these reagents (data not shown), suggesting that duck keep bone resorption under tight control and at substantially lower levels as a means to lengthen their beaks. Taken together, our ability to manipulate beak length in quail by modulating resorption is congruent with our two other independent lines of evidence showing that quail have more TRAP activity (in whole mount and in section), and higher expression of *Mmp13* and *Mmp9* (via RT-qPCR and *in situ* hybridization). Thus, the species-specific regulation of bone resorption by NCM appears to be a key mechanism establishing beak length.

### 3.4. NCM establishes species-specific bone mineral density

**Fig. 5:**
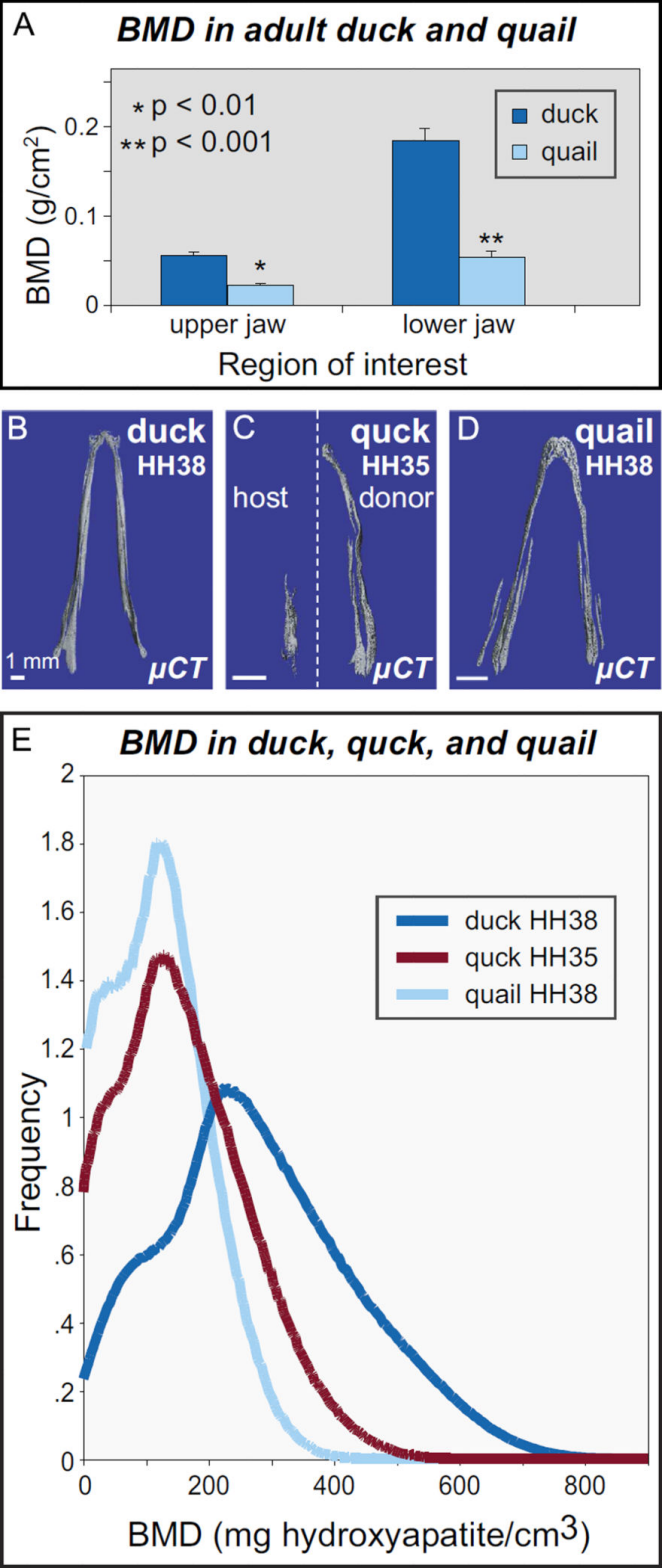
NCM regulates bone mineral density. (A) Duck jaw bones (*n*=3) have higher BMD than do quail (*n*=3). (B) µCT reconstructions of mineralized bone in lower jaws for duck at HH38, (C) quck at HH35, and (D) quail at HH38. (E) µCT histogram of BMD in lower jaws of HH38 quail (*n*=2, light blue), donor-side HH35 quck (*n*=3, red) and HH38 duck (*n*=3, dark blue). Duck have a higher average BMD than quail, and quck are quail-like.

Due to the well-known role of osteocytes and osteoclasts in the regulation of bone mineral density (BMD), we also investigated whether as a consequence of NCM-mediated differences in bone resorption there exist species-specific differences in BMD that relate to the functional morphology of the beak. For example, in aquatic species, a higher BMD enables negative buoyancy during diving (Wall, 1983). We used dual energy X-ray absorptiometry (DXA) and µCT to measure BMD in upper and lower aspects of the beak skeleton in adult and embryonic quail and duck. In adult duck, we find a higher BMD, and thus, more mineralized tissue compared to that of quail (Fig. 5(A)). In 3D reconstructions, we find that there is more bone in the duck jaw and that this bone is more highly mineralized and smoother than in quail (Fig. 5(B,D)). Qualitatively, in quck at HH35, bone on the donor side appeared comparable to that of HH38 quail likely due to the accelerated maturation rate of quail donor NCM. Quantitatively, HH38 quail had an average BMD across the lower beak of about 180 mg hydroxyapatite/cm^3^, while duck had a BMD distribution averaging about 300 mg hydroxyapatite/cm^3^. In HH35 quck, the distribution was similar to that of HH38 quail, with an average around 190 mg hydroxyapatite/cm^3^ (Fig. 5(E)).

Thus, the capacity of NCM to regulate bone resorption not only affects bone length but also BMD in the beak skeleton. This is striking, given that duck are waterfowl and use their elongated and denser beaks for digging around in sediment, and as a countermeasure to overcome buoyancy issues (*e.g.*, air trapped in plumage) while dipping their heads beneath water to capture prey items or when diving. Accordingly, our study implies that control of bone resorption by NCM is a crucial mechanism enabling such adaptations to be acquired during evolution.

## 4. Conclusion

A wide range of genetic and embryological studies have shown that the establishment of beak pattern is a complex process involving numerous gene regulatory networks, reciprocal signaling interactions, and hierarchical levels of control. During the past decade, several molecules have been identified that are differentially expressed at the right time and place to account for the evolution of beak pattern among morphologically distinct birds. For example, discrete spatial domains of *Bmp4* in beak precursor cells correspond to species-specific variations in beak depth and width among Darwin’s finches, chicks, ducks, and cockatiels (Abzhanov et al., 2004; Wu et al., 2006, 2004). Similarly, secreted factors such as SHH, FGFs, WNTs, and BMPs that emanate from adjacent epithelial tissues have also been shown to play a role in mediating the shape and outgrowth of the beak (Abzhanov and Tabin, 2004; Ashique et al., 2002; Bhullar et al., 2015; Brugmann et al., 2007; Brugmann et al., 2010; Doufexi and Mina, 2008; Foppiano et al., 2007; Grant et al., 2006; Havens et al., 2008; Hu and Marcucio, 2009, 2012; Hu et al., 2015a, 2015b; MacDonald et al., 2004; Mina et al., 2002; Richman et al., 1997; Rowe et al., 1992; Schneider et al., 2001; Szabo-Rogers et al., 2008; Wu et al., 2006; Young et al., 2014). But exactly how these pathways are regulated by NCM and how changes to their regulation alter the dimensions of the beak have remained unclear. Most analyses in this regard, have focused on the differential proliferation of NCM, as well as on the deposition of bone and cartilage. In contrast, our current work uncovers a novel function for bone resorption, which is to help establish species-specific jaw length; and our transplant experiments indicate that the underlying molecular mechanisms stem from the capacity of NCM to control the activity of its own derivatives (i.e., osteocytes) and also that of mesoderm-derived osteoclasts.

Such results complement and build upon previous studies on Darwin’s finches and other species, which argue that an important regulator of beak length is the calcium binding protein, *calmodulin* (Abzhanov et al., 2006; Gunter et al., 2014; Schneider, 2007). The calmodulin-dependent pathway is known to control osteocytes and osteoclasts locally (Choi et al., 2013a, 2013b; Seales et al., 2006; Zayzafoon, 2006) and our work shows that the actions of osteocytes and osteoclasts during resorption are not only mediated by NCM, but that they are also a critical component of species-specific programs for beak length. Thus, our results support the notion that calcium regulation may serve as an important mechanism for evolvability generally (Kirschner and Gerhart, 1998), and for establishing jaw length more specifically (Gunter et al., 2014; Parsons and Albertson, 2009), with calmodulin functioning in birds during craniofacial pattern formation at early stages (around HH26) and resorption being essential during beak growth at later stages (HH34 onwards) through the activity of molecules like MMPs. Moreover, in this context it is tempting to speculate that resorption may be a fundamental mechanism during beak evolution that is especially responsive to the availability and intake of dietary calcium in diverse ecological niches, the endocrine effects of calcium-dependent hormones, and the local and tissue-specific modulation of calcium signaling within the developing beak primordia (Schneider, 2007).

In the context of our published work as well as that of others, the use of the quail-duck chimeric system has allowed us to discern a variety of discrete mechanisms that operate through three phases of development and enable NCM to establish jaw length (Schneider, 2015). First, during the early regionalization of the neural tube, duck have a broader midbrain from which NCM-derived jaw progenitors emigrate, and this endows duck with about 15% more initial cells to form the jaw skeleton (Fish et al., 2014). Second, as these jaw progenitors expand, there is NCM-mediated species-specific control over the cell cycle, which quickly doubles the size of the duck jaw primordia relative to stage-matched quail. NCM appears to accomplish this feat by differentially regulating and responding to members and targets of the SHH, FGF, BMP, and TGFβ pathways in a species-specific manner, and by executing autonomous molecular and cellular programs for the formation of cartilage and bone through spatial and temporal patterns of gene expression that are intrinsic to each species (Ealba and Schneider, 2013; Eames and Schneider, 2008; Fish and Schneider, 2014b; Hall et al., 2014; Jheon and Schneider, 2009; Merrill et al., 2008; Mitgutsch et al., 2011; Schneider and Helms, 2003). For example, by the time the jaw skeleton becomes mineralized, *Runx2* levels in quail are more than double those of duck (Hall et al., 2014). Experimentally increasing levels of *Runx2* in chick embryos dramatically decreases the size of the beak skeleton (Eames et al., 2004; Hall et al., 2014), which parallels the predicted relationship between *Runx2* expression levels and facial length described for adult dogs and other mammals based on numbers of tandem repeats (Fondon and Garner, 2004; Pointer et al., 2012; Sears et al., 2007). Thus, another mechanism that affects jaw length is the way NCM exerts precise control over the expression levels of key transcription factors and the timing of skeletal cell differentiation.

Finally, the current study has shed light on a third mechanism that influences jaw length by demonstrating that the amount of bone resorption in birds is inversely proportional to beak length, that bone resorption is NCM-mediated, and that modulating bone resorption can lengthen or shorten the beak. These findings are consistent with prior studies invoking differential fields of resorption to explain how changes in size and shape occur in the developing jaw skeleton of humans (Enlow et al., 1975; Moore, 1981; Radlanski and Klarkowski, 2001; Radlanski et al., 2004). Our results may also help clarify the etiology of the altered snouts of mice with mutations in genes known to affect resorption such as *Mmp2* (Egeblad et al., 2007), and jaw length defects in humans with conditions such as Juvenile Paget’s disease (i.e., *Opg*) and Spondyloepimetaphyseal dysplasia (i.e., *Mmp13*) (Gorlin et al., 1990; Lezot et al., 2014). Moreover, our experiments suggest that precisely targeted pharmacological approaches that carefully modulate bone resorption might one day be used to treat human defects in jaw length such as mandibular hypoplasia or malocclusion. Such a goal is supported by other work demonstrating that bisphosphonates can alter mandibular growth in mice and rats (Kimura et al., 2008; Lezot et al., 2014; Oyhanart et al., 2015). We conclude that the proclivity of NCM to maintain spatiotemporal control over the induction, differentiation, deposition, mineralization, and resorption of bone is what integrates the determinants of jaw length throughout development, and is what empowers NCM with its inimitable ability to generate variation during disease and evolution.

## Figures and Tables

**Table 1:** Quantification of bone deposition as represented by osteoid staining and bone resorption as indicated by TRAP staining in the mandibles of duck (*n*=4), quail (*n*=4), and the quail donor side versus duck host side of chimeric quck (*n*=2). Values shown are the mean volumes for each group with their standard deviations.

	Duck mandible	Quail mandible	Quck donor mandible	Quck host mandible
Osteoid volume (mm^3^)	0.212 ± 0.055	0.048 ± 0.01	0.167 ± 0.018	0.162 ± 0.026
TRAP volume (mm^3^)	0.002 ± 0.002	0.028 ± 0.009	0.040 ± 0.017	0.008 ± 0.008
